# Purification and Characterization of a Novel Kazal-Type Trypsin Inhibitor from the Leech of *Hirudinaria manillensis*

**DOI:** 10.3390/toxins8080229

**Published:** 2016-07-23

**Authors:** Yanmei Lai, Bowen Li, Weihui Liu, Gan Wang, Canwei Du, Rose Ombati, Ren Lai, Chengbo Long, Hongyuan Li

**Affiliations:** 1 Department of Endocrine and breast Surgery, The First Affiliated Hospital of Chongqing Medical University, Chongqing 400016, China; yanmay_lai@163.com; 2 Key Laboratory of Animal Models and Human Disease Mechanisms of Chinese Academy of Sciences & Yunnan Province, Kunming Institute of Zoology, Kunming 650223, China; lbw19901006@126.com (B.L.); liuweihui00@126.com (W.L.); wang.gan@outlook.com (G.W.); 3 Life Sciences College, Nanjing Agricultural University, Nanjing 210095, China; ducw1992@163.com; 4 Sino-Africa Joint Research Center, Chinese Academy of Sciences, Kunming Institute of Zoology, Kunming 650223, Yunnan, China; roseombati@gmail.com; 5 Institute of Primate Research, P.O Box 24481, Nairobi, Kenya

**Keywords:** *Hirudinaria manillensis*, Kazal-type trypsin inhibitor, bdellin, protease inhibitor

## Abstract

Kazal-type serine proteinase inhibitors are found in a large number of living organisms and play crucial roles in various biological and physiological processes. Although some Kazal-type serine protease inhibitors have been identified in leeches, none has been reported from *Hirudinaria manillensis*, which is a medically important leech. In this study, a novel Kazal-type trypsin inhibitor was isolated from leech *H. manillensis*, purified and named as bdellin-HM based on the sequence similarity with bdellin-KL and bdellin B-3. Structural analysis revealed that bdellin-HM was a 17,432.8 Da protein and comprised of 149 amino acid residues with six cysteines forming three intra-molecular disulfide bonds. Bdellin-HM showed similarity with the Kazal-type domain and may belong to the group of “non-classical” Kazal inhibitors according to its Cys^I^-Cys^II^ disulfide bridge position. Bdellin-HM had no inhibitory effect on elastase, chymotrypsin, kallikrein, Factor (F) XIIa, FXIa, FXa, thrombin and plasmin, but it showed a potent ability to inhibit trypsin with an inhibition constant (*K_i_*) of (8.12 ± 0.18) × 10^−9^ M. These results suggest that bdellin-HM from the leech of *H. manillensis* plays a potent and specific inhibitory role towards trypsin.

## 1. Introduction

Protease inhibitors are ubiquitously distributed in living organisms such as plants, animals, fungi, and bacteria, which have the ability to target proteinase through different mechanisms resulting in either complete or partial inhibition. A large number of studies suggest that protease inhibitors have promising therapeutic uses in the treatment of many diseases [1,2,3,4,5,6,7,8]. According to the types of proteases, protease inhibitors are mainly classified into four classes: serine, cysteine, aspartic and metalloproteinase inhibitors [9]. The Kazal family of protease inhibitors is one of the best-known serine protease inhibitor families [10].

Kazal-type serine proteinase inhibitors are named according to the function of pancreatic secretory trypsin inhibitor (SPINK1) first isolated by Kazal et al. [11]. They still assist numerous physiological processes including food digestion, wound healing, innate immunity and protection against proteolytic enzymes [12]. All of the Kazal-type serine proteinase inhibitors contain single or multiple Kazal inhibitory domains linked together by peptide spacers of variable length [13]. The Kazal-type inhibitory domains can be classified as typical or atypical Kazal domains. Generally, typical Kazal domains have six cysteine residues forming a 1–5, 2–4, 3–6 disulfide bond pattern [14,15], while atypical Kazal domains only have two disulfide bonds [15,16,17]. When they react with proteases, the P_1_ residue described as residing at the second amino acid downstream of the second conserved cysteine residue is inserted into the S_1_ specificity pocket of the protease in a substrate-like manner [18]. In addition, based on the values of *m* and *n* where the subscripts *m* and *n* are integral numbers of amino acid residues in the frameworks Cys^I^-(X)*_m_*-Cys^II^ and Cys^IV^-(X)*_n_*-Cys^V^, classical and non-classical Kazal domains have been defined [19]. The Kazal-type serine proteinase inhibitors possess highly homologous three-dimensional structures despite the differences in the length of amino acid sequences between the cysteines and amino acid sequence variation [20].

In order to take a blood meal and acclimatize themselves to variable environments, the blood-sucking animals have evolved potent chemical defense systems including a variety of protease inhibitors to eliminate the proteolytic enzymes from biological systems. A large number of protease inhibitors were identified from leeches of medical importance. They are: bdellastasin (inhibitor of trypsin and plasmin), hirustasin (inhibitor of tissue kallikrein, trypsin, α-chymotrypsin, and granulocyte cathepsin G), eglin (inhibitor of α-chymotrypsin, subtilisin, and chymosin and the granulocyte proteinases elastase and cathepsin G) hirudin (thrombin inhibitor) from *H. medicinalis* [21], guamerin (inhibitor of elastase and chymotrypsin) and bdellin-KL (inhibitor of trypsin, plasmin and acrosin) from *H. nipponia* [22,23]. Some of these belong to Kazal-type serine proteinase inhibitors.

Although some Kazal-type serine proteinase inhibitors have been identified in leeches, to date none has been reported from *H. manillensis*, a leech more specialized in feeding on mammalian blood. In this study, bdellin-HM was purified and characterized from the head of leech *H. manillensis*. Here, based on our results, bdellin-HM shows a potent and specific inhibitory effect on trypsin. To the best of our knowledge, this is the first report about a Kazal-type serine protease inhibitor from *H. manillensis*.

## 2. Results

### 2.1. Purification of Bdellin-HM

**Figure 1:**
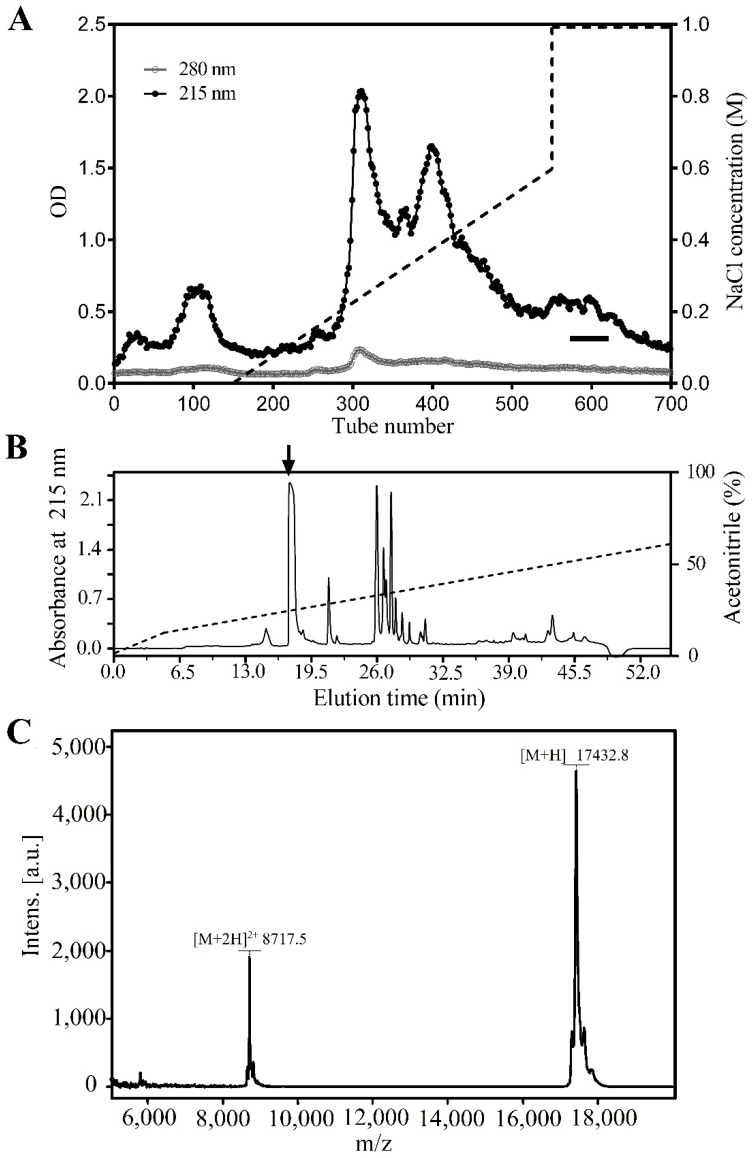
Purification of bdellin-HM from *H. manillensis*. (**A**) Crude extracts were fractionated using DEAE Sephadex A-50 ion exchange. The fractions with antitrypsin activity were indicated by a bar; (**B**) The antitrypsin fractions were further purified by C_18_ reverse-phase high-performance liquid chromatography (RP-HPLC). The protein peak containing activity to inhibit trypsin was marked by an arrow; (**C**) matrix-assisted laser desorption ionization time-of-flight (MALDI-TOF) analysis of purified native dbellin-HM.

The crude extracts of *H. manillensis* were resolved into several fractions by DEAE Sephadex A-50 column. The fraction with trypsin inhibitory activity is indicated by a bar (Figure 1A) and then was applied to a C_18_ RP-HPLC column for further purification. The peptide (marked by an arrow) containing antitrypsin activity named bdellin-HM was purified (Figure 1B). MALDI-TOF-MS analysis gave an observed molecular weight (MW) of 17,432.8 Da (Figure 1C) by using a positive ion and linear mode, with specific operating parameters including a 20 kV ion acceleration voltage, 50-time accumulation for single scanning, and ±0.1% accuracy of mass determinations.

### 2.2. Primary Structure of Bdellin-HM

**Figure 2:**
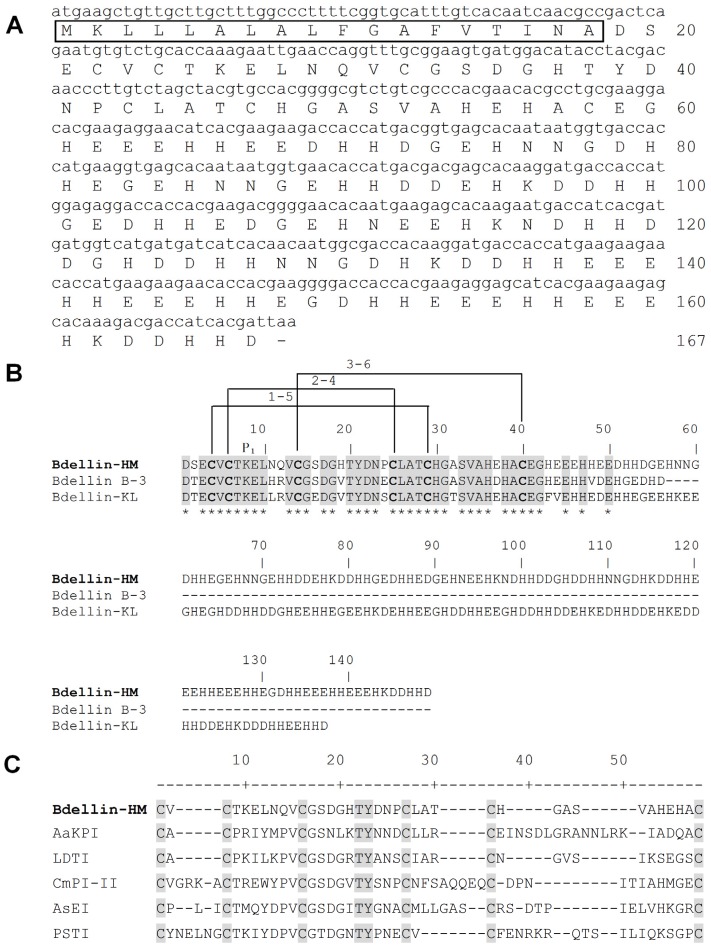
cDNA sequence encoding bdellin-HM precursor and sequence comparison with other leech protease inhibitors. (**A**) The nucleotide sequence encoding bdellin-HM precursor and the deduced amino acid sequence. The bar (-) indicates stop codon. The signal peptide is boxed; (**B**) The sequence comparison of bdellin-HM with bdellin B-3 and bdellin-KL. The identical amino acid residues are indicated by an asterisk (*); (**C**) Multiple sequence alignment of the Kazal domain from *H. manillensi* (Bdellin-HM), *H. medicinalis* (LDTI P80424), *Aedes aegypti* (AaKPI ABF18209), *Cenchritis muricatus* (CmPI-II P84755), *Anemonia sulcata* (AsEI 1Y1B) and *Homo sapiens* (PSTI P00995). The conserved threonine-tyrosine residues between cysteine 3 and 4 are indicated. They are found to contain the same cysteine motifs.

**Figure 3:**
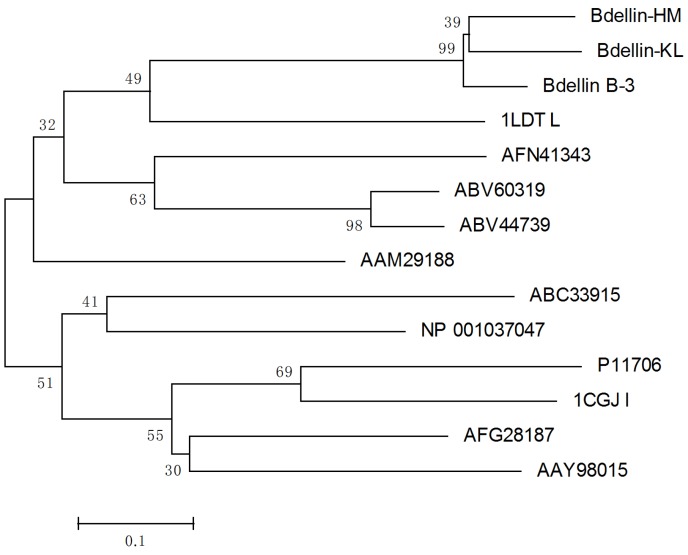
Phylogenetic analysis of bdellin-HM and other kazal-type serine protease inhibitors’ amino acid sequences based on the neighbor-joining method by using MEGA 5.1. The origin of amino acid sequences and their GenBank accession numbers are as follows: Bdellin-KL: *H. nipponia* (AAF73890); Bdellin B-3: *H. medicinalis* (P09865); *H. medicinalis* (1LDT_L); *Culex pipiens pallens* (AFN41343); *Lutzomyia longipalpis* (ABV60319); *Phlebotomus papatasi* (ABV44739); *Neospora caninum* (AAM29188); *Fenneropenaeus chinensis* (ABC33915); *Bombyx mori* (NP_001037047); *Anguilla anguilla* (P11706); *Homo sapiens* (1CGJ_I); *Glossina morsitans morsitans* (AFG28187); *Stomoxys calcitrans* (AAY98015).

The partial *N*-terminal sequence of bdellin-HM was determined as DSECVCTKELNQVCGSDGHTYDNPC by automatic Edman degradation. According to the *N*-terminal sequence, degenerate primers were designed (Table 1) to clone the cDNA encoding the precursor of bdellin-HM from the cDNA library. A 504-bp cDNA encoding the precursor of bdellin-HM was obtained. The cDNA has an open reading frame of 501 nucleotides coding a pro-protein of 167 amino acids including a signal peptide of 18 residues and mature bdellin-HM of 149 residues (Figure 2A). A Blast search shows bdellin-HM is similar to bdellin B-3 [24] and bdellin-KL [23] which are Kazal-type serine protease inhibitors (Figure 2B). The theoretical MW of linear mature bdellin-HM was calculated as 17,438.04 Da by http://web.expasy.org/compute_pi/. Considering that bdellin-HM contains six cysteines, which are likely to form three intra-molecular disulfide bridges and a typical Kazal domain, its MW should be 17,432.04 Da, which matches well with the observed molecular weight 17,432.8 Da (Figure 1C) determined by MALDI-TOF-MS. The analysis of the multiple sequence alignment showed that six cysteine residues and the threonine-tyrosine residues are highly conserved among different species (Figure 2C). Phylogenetic analysis of bdellin-HM with homologs in 13 other species using their mature peptide region sequences indicated that bdellin-HM showed close genetic distances with bdellin-KL (Figure 3).

### 2.3. Protease Inhibitory Activity and Enzyme Kinetics

**Figure 4:**
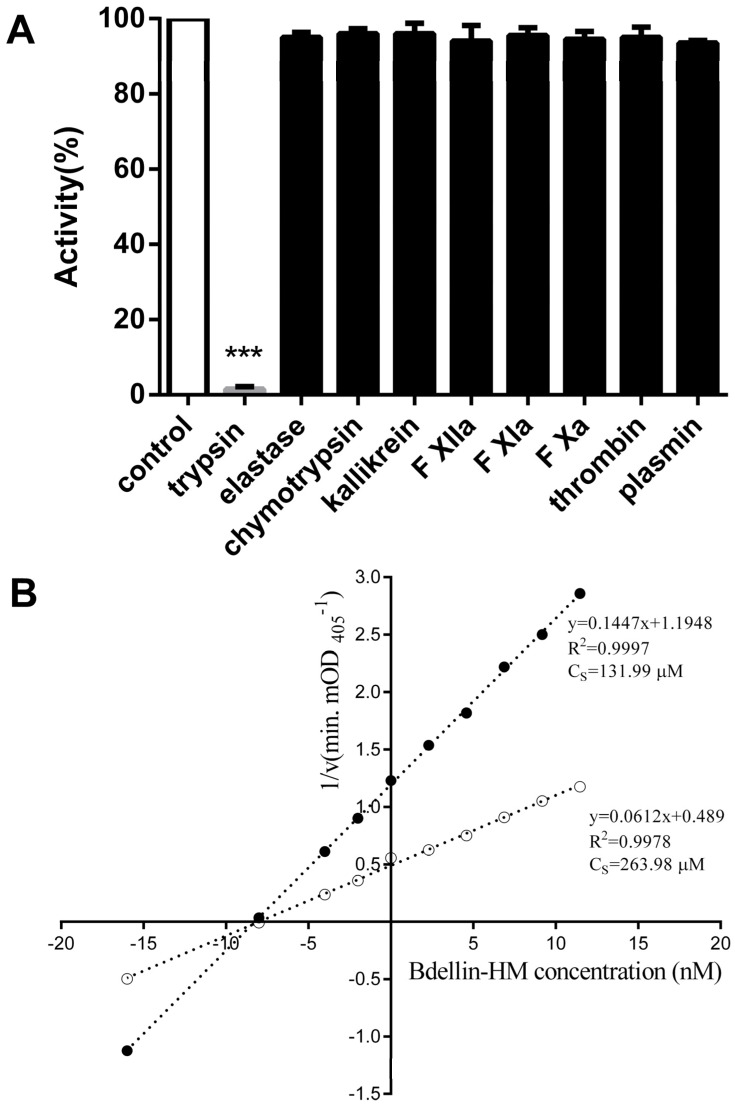
Effects of bdellin-HM on proteases. (**A**) Enzymes were incubated with bdellin-HM (11.5 μM), and catalytic activity was estimated by chromogenic substrate hydrolysis. Values are expressed as mean ± SD (*n* = 4). *** *p* < 0.001 compared with the control group; (**B**) Bdellin-HM was found to be a competitive inhibitor with an inhibition constant (*K_i_*) of (8.12 ± 0.18) × 10^−9^ M determined by the method of Dixon.

Under the assay conditions, bdellin-HM inhibited the activity of trypsin (bovine pancreas) potently, whereas no inhibitory activity on elastase, chymotrypsin, kallikrein, FXIIa, FXIa, FXa, thrombin and plasmin was observed (Figure 4A). Enzyme kinetic study showed that bdellin-HM was a competitive inhibitor with an inhibition constant (*K_i_*) of (8.12 ± 0.18) × 10^−9^ M (Figure 4B).

## 3. Discussion

The medically important leech has been used as a traditional treatment for chronic diseases for more than 2000 years. A considerable quantity of trypsin-plasmin inhibitors known as bdellins have been found in the salivary glands as well as in other organs of the leech, *H. medicinalis* [25,26]. Bdellin B-3, one of these, was a single-domain Kazal inhibitor [24]. In addition, a potent trypsin-plasmin inhibitor-bdellin-KL sharing similar amino acid sequence to bdellin B-3 was reported from *H. nipponia* [23]. *H. manillensis* belongs to the same order Arynchobdellida as *H. medicinalis* and it is significantly more specialized for feeding on mammalian blood [27]. In this report, a novel Kazal-type trypsin inhibitor named bdellin-HM was isolated from the head of *H. manillensis* and further characterized (Figure 1). The cDNA encoding bdellin-HM precursor was cloned from the cDNA library. Mature bdellin-HM is composed of 149 amino acid residues (Figure 2A). It shows high similarity to bdellin B-3 and bdellin-KL by sequence analysis (Figure 2B). Similar to bdellin B-3 and bdellin-KL, bdellin-HM also has six cysteine residues which can form three disulfide bonds and belongs to the class of typical Kazal domains. According to the number of amino acid residues between the cysteine residues, Kazal-type domains are divided into classical and non-classical Kazal domains [28]. Only one amino acid residue is between the first and second cysteine in bdellin-HM, indicating that it belongs to the family of non-classical Kazal domains.

Bdellin-HM is a competitive trypsin inhibitor with an inhibition constant (*K_i_*) of (8.12 ± 0.18) × 10^−9^ M, but no inhibitory effects on elastase, chymotrypsin, kallikrein, FXIIa, FXIa, FXa, thrombin or plasmin were observed under the assay conditions (Figure 4). Bdellin B-3 and bdellin-KL block the activity of trypsin with the dissociation constant of <10^−10^ M [29] and (1.36 ± 0.42) × 10^−9^ M [23] respectively. The main role of Kazal-type serine proteinase inhibitors is to limit and regulate the spread of serine proteinase activity [30].

The mechanism of the Kazal-type inhibitor complies with the ‘standard’ mechanism, where the active loop on the inhibitor binds to the active site of the enzyme in a substrate enzyme-like manner [12]. The inhibitory specificity of a Kazal domain is governed by the P_1_ amino acid residue described as residing at the second amino acid downstream of the second conserved cysteine residue. Generally, if the P_1_ amino acid is Lys or Arg, the Kazal-type serine proteinase inhibitor inhibits trypsin strongly. The P_1_ amino acid (the eighth amino acid) of bdellin B-3, bdellin-KL and bdellin-HM is the same Lys, but there are different functions. Bdellin B-3 and bdellin-KL are able to inhibit trypsin and plasmin [23,29], while bdellin-HM has an inhibitory effect on trypsin only. This may be due to the different amino acid residues in other contact positions. Contact positions which were mentioned by several structural studies can influence the efficiency as well as specificity of the combination between Kazal domains and proteases, although the inhibitory specificity is determined mainly by the P_1_ [12,31]. Twelve contact positions are reported, namely: P_6_, P_5_, P_4_, P_3_, P_2_, P_1_, P_1_′, P_2_′, P_3_′, P_14_′, P_15_′ and P_18_′, where the numerals description starts from P_1_, P_2_, P_3_… to the *N*-terminus and P_1_′, P_2_′, P_3_′… to the *C*-terminus [32]. Multiple alignments reveal that P_3_′ is His (basic residue), Asn (other residue) and Leu (hydrophobic uncharged residue) in bdellin B-3, bdellin-HM and bdellin-KL, respectively. In addition, the P_4_′ residue of bdellin-HM is Gln, which is different from Arg in the same position of bdellin B-3 and bdellin-KL. Compared with bdellin B-3 and bdellin-KL, different residues in P_3_′ and P_4_′ positions of bdellin-HM may contribute to its effects on proteases. Further research such as site-directed mutagenesis should be performed to address these questions.

## 4. Conclusions

In conclusion, bdellin-HM, a 17,432.8-Da protein, was for the first time purified from the head of *H. manillensis* by DEAE Sephadex A-50 ion exchange, RP-HPLC and MALDI-TOF analysis. It was found to possess the characteristic of Kazal-type serine protease inhibitors and showed no inhibitory activity on elastase, chymotrypsin, kallikrein, FXIIa, FXIa, FXa, thrombin and plasmin under the assay conditions. However, Enzyme kinetic study proved that bdellin-HM was a competitive inhibitor with an inhibition constant (*K_i_*) of (8.12 ± 0.18) × 10^−9^ M.

## 5. Materials and Methods

### 5.1. Collection of Crude Extracts

*H. manillensis* leeches were purchased from Guangxi Province of China. The leeches were transported to the laboratory still alive. Crude extracts were prepared from the head part of the leeches as described previously [33]. In brief, leech heads were dissected out from bodies, washed in 0.9% saline and quickly frozen and then grounded within liquid nitrogen.

### 5.2. Purification of Bdellin-HM

The crude extracts were lyophilized and dissolved in 50 mM Tris-HCl buffer, pH 8.9. Subsequently, they were loaded on a DEAE Sephadex A-50 column (GE Healthcare Life Sciences, Chicago, IL, USA, 5 cm diameter, 60 cm length) that was previously equilibrated with the same buffer. Sample fractionation was carried out by eluting the column with a linear gradient of NaCl. Elution was performed with a flow rate of 1.5 mL/min at 4 °C, and fractions were collected in each tube containing 15.0 mL. The absorbance of the elution fractions was monitored at both 215 and 280 nm. Fractions with trypsin inhibitory activity were pooled and lyophilized prior to further purification. The fraction from the previous step was resuspended and applied to reverse-phase high-performance liquid chromatography (RP-HPLC) on a C_18_ column (Waters, Milford, MA, USA, 5 μm particle size, 250 × 4.6 mm). Elution was carried out with a linear gradient of 10%–60% solution B (99.9% acetonitrile, 0.1% TFA) for 60 min at a flow rate of 1 mL/min. The eluted fraction containing antitrypsin activity was collected.

### 5.3. Mass Spectrometric Analysis and Sequencing of Peptide

The molecular weight of purified native protein was analyzed by matrix-assisted laser desorption ionization time-of-flight mass spectrometry (MALDI-TOF-MS, AXIMA CFR, Kratos Analytical, Shimadzu Corporation, Kyoto, Japan). Partial peptide sequence was determined by automatic Edman degradation on a pulsed liquid-phase sequencer (Applied ProciseTM 491, Shimadzu Corporation, Kyoto, Japan).

### 5.4. RNA Extraction and cDNA Library Construction

Total RNA was extracted from the head of *H. manillensis* using Trizol Reagent (Life Technologies, Carlsbad, CA, USA) according to the manufacturer’s instructions. The SMART™ PCR cDNA Construction kit (Clontech, Palo Alto, CA, USA) was used for synthesizing cDNA as described previously [34].

### 5.5. Screening of cDNA Encoding Bdellin-HM

To screen the cDNA encoding the precursor of bdellin-HM, the synthesized cDNA was used as the template for PCR. Two pairs of oligonucleotide primers (Table 1) were used in PCR reactions, where primers 1 and 3 were designed according to the partial *N*-terminal sequence of bdellin-HM determined by Edman degradation and primers 2 and 4 are from the SMART™ (Clontech, Palo Alto, CA, USA) PCR cDNA Construction kit. The PCR conditions were 5 min at 95 °C, and then running 30 cycles with a temperature of 30 s at 95 °C, 30 s at 60 °C, and 60 s at 72 °C, finally holding at 72 °C for 10 min. The final PCR products were cloned into pGEM^®^-T Easy vector (Promega, Madison, WI, USA). An Applied Biosystems DNA sequencer (Model ABI PRISM 377, Shimadzu Corporation, Kyoto, Japan) was used for DNA sequencing. Subsequently, the blast search (NCBI) was performed for sequence alignment with other previously reported peptides. Multiple sequence alignments were performed by DNAman software (Lynnon Biosoft, Quebec, QC, Canada). The phylogenetic tree was constructed by using MEGA 5.1 (Tokyo Metropolitan University, Tokyo, Japan) with the neighbor-joining (NJ) method.

### 5.6. Protease Inhibitory Assays and Enzyme Kinetic Study

The protease inhibitory assays of bdellin-HM were performed in 10 mM HEPES, pH 7.4 buffer containing 150 mM NaCl, 3 mM EDTA and 0.05% *v*/*v* Surfactant P_20_ at 37 °C, with a final volume of 100 μL. The proteases and their respective chromogenic substrates used in the tests include 0.2 mM urokinase and plasmin substrate (Gly-Arg-*p*-nitroanilide dihydrochloride, SIGMA, St. Louis, MO, USA) for 400 nM bovine pancreas trypsin and plasmin (SIGMA), 0.2 mM *N*-Methoxysuccinyl-Ala-Ala-Pro-Val-*p*-nitroanilide (SIGMA) for 400 nM elastase (SIGMA), 0.2 mM *N*-Succinyl-Gly-Gly-Phe-*p*-nitroanilide (SIGMA) for 400 nM chymotrypsin (SIGMA), 0.2 mM H-d-Phe-Pip-Arg-*p*NA.2HCl (Hyphen Biomed, Neuville-sur-Oise, France) for 10 nM thrombin (SIGMA), 0.2 mM H-d-Pro-Phe-Arg-*p*NA.2HCl (Hyphen Biomed) for 400 nM Kallikrein, 400 nM FXIa and 10 nM FXIIa (Enzyme Research Laboratories, South Bend, IN, USA), and 0.2 mM CH3OCO-D-CHA-Gly-Arg-*p*NA-AcOH (SIGMA) for 10 nM FXa (Enzyme Research Laboratories). The proteases were separately pre-incubated with bdellin-HM (final concentration of 11.5 μM) for 5 min at 37 °C. The reactions were started by the addition of the corresponding chromogenic substrate and immediately the absorbance of formed *p*-nitroaniline was measured at 405 nm every 30 s for 30 min by an Epoch microplate spectrophotometer (Bio-Tek, Winooski, VT, USA). Enzyme kinetic study of bdellin-KM was performed by using different concentrations of bdellin-HM (0, 2.3, 4.6, 6.9, 9.2, and 11.5 nM) and Gly-Arg-*p*-nitroanilide dihydrochloride (131.99 and 263.98 μM). Finally, the method of Dixon was used to determine the inhibition constant (*K_i_*) value of bdellin-HM on trypsin.

## Figures and Tables

**Table 1:** The primers used for cDNA cloning of bdellin-HM.

Primer	Sequence
1	5′-GAYWSNGARTGYGTNTGYAC-3′
2	5′-AAGCAGTGGTATCAACGCAGAGT-3′
3	5′-CTYACRCANACRTGNTTY-3′
4	5′-ATTCTAGAGGCCGAGGCGGCCGA-3′

Primers 1 and 2 for mature peptide cloning; primers 3 and 4 for signal peptide cloning. (R = A/G; Y = C/T; S = C/G; W = A/T; N = A/C/G/T).
